# Data leakage in machine learning studies creep into meta-analytic estimates of predictive performance

**DOI:** 10.1038/s41380-025-03336-y

**Published:** 2025-10-30

**Authors:** Laurens A. van de Mortel, Guido A. van Wingen

**Affiliations:** 1 Amsterdam UMC, location University of Amsterdam, Department of Psychiatry, Meibergdreef 9, Amsterdam, The Netherlands; 2 Amsterdam Neuroscience, Amsterdam, The Netherlands

**Keywords:** Predictive markers, Neuroscience

To the Editor,

We read with great interest the article by Long et al. (2024) titled *“Predicting treatment outcomes in major depressive disorder using brain magnetic resonance imaging: a meta-analysis”*. In this article, the authors compare the accuracy of MRI and clinical data to predict treatment outcomes for major depressive disorder (MDD). By grouping studies using brain MRI and clinical variables separately across different MDD treatments and outcome definitions, the authors report a statistically significant higher log Diagnostic Odds Ratio (logDOR) for brain MRI (2.53) compared to clinical variables (1.62). We agree that the use of brain MRI to improve treatment efficacy and guiding personalized treatment selection is an exciting and promising prospect, and comparing its added value over readily available clinical data is important and a clinically relevant question.

However, upon closer inspection of the included studies, we found that many suffer from a methodological flaw known as *data leakage*. This occurs when variables showing statistically significant group-level differences (e.g., between responders and non-responders) are subsequently used to train machine learning models to predict those same outcomes in the entire dataset. This procedure reuses outcome-related information from the test set during model building. Such circularity is well known to inflate apparent prediction accuracies and limit true generalizability to unseen data [1–4], often producing performance estimates that do not replicate in independent samples.

We identified at least 20 MRI studies (45%) and 5 clinical studies (38%) in which the manuscript reported procedures that are consistent with this phenomenon (see Supplementary Table 1). These studies are therefore likely to overestimate their predictive performance. Additionally, three studies combined MRI and clinical data into a single model, making it impossible to determine the predictive value of MRI features alone.

To assess the impact of these issues, we re-estimated the average log(DOR) for both MRI and clinical studies, including only those studies that, to the best of our assessment, used independent data to evaluate predictive performance. Our re-analysis was conducted in RStudio using the *metafor* [5] package using the log(DOR) values reported in the study by Long et al. Average log(DOR) values decreased for both clinical (from 1.62 to 1.32) and MRI studies (from 2.53 to 2.02) after excluding the studies with apparent data leakage. MRI-based models still showed a statistically higher log(DOR) than clinical models, albeit with a p-value of 0.04 and a much higher heterogeneity observed among studies than initially reported (see Supplementary Figure 1).

Although our analytic approach differs slightly from that of Long et al. and significance levels may vary under different specifications, these results suggest that MRI may still outperform clinical measures in predicting treatment outcome in MDD, but that the advantage is much smaller and less certain than the authors originally reported. Importantly, many of the retained MRI studies also relied on leave-one-out cross-validation (LOOCV). While LOOCV can be valuable in small datasets, it is prone to high variance and, in this context, can overestimate model performance [6]. This limitation, combined with the prevalence of data leakage in the literature, highlights the urgent need for larger, prospectively designed studies that use fully independent validation data, preregistration, and transparent reporting of analytical pipelines [7].

We commend the authors of the study for tackling an important question and for their large body of work. Nevertheless, the high percentage of peer-reviewed studies with apparent data leakage and our findings underscore the importance of scrutinizing methodological quality in meta-analyses of predictive modeling studies. When performing such analyses, quality assessment tools such as the REFORMS and PROBAST-AI questionnaires should be used to assess methodological biases in selected studies [8, 9]. Without careful exclusion of studies with high risk of bias, pooled performance estimates risk overestimating the true predictive value of brain MRI in clinical practice and paints an overly optimistic picture. Though even after excluding studies with apparent data leakage, the pooled predictive performance estimates are still impressive from the perspective that clinicians perform at chance level [10].

Ultimately, the goal of predictive neuroimaging in MDD should be to provide reliable, generalizable tools that can meaningfully inform treatment decisions. Achieving this will require not only advances in neuroimaging technology and machine learning methods but also rigorous methodological standards in both primary studies and meta-analyses.

## Supplementary information

Supplementary Table 1

Supplementary Figure 1
