# Supplementary material for: Data leakage in machine learning studies creep into meta-analytic estimates of predictive performance
Source: Mol Psychiatry. 2025 Oct 30;30(12):6070–1. doi: 10.1038/s41380-025-03336-y (PMC12602356; doi:10.1038/s41380-025-03336-y)
Supplement: Supplementary file 1 — Supplementary Table 1 [file 41380_2025_3336_MOESM1_ESM.docx]

Supplementary Table 1: Excluded studies for both the clinical and MRI data and the reasons for exclusion (direct quotes from article where available).

| Excluded Study | Exclusion Reason |
| --- | --- |
| Zhou et al., 2022 | Clinically significant features in group-level analysis are used for ROC analysis |
| Wagner et al., 2017 | *"In a first step, differences between improver and non-improver in the investigated predictors (s. Table 1) were analysed using t-tests for independent variables or Chi2-tests in order to identify variables with significant differences between early improver and non-improver (data not shown, Supplementary Tables 1 and 2). In a second step, significant variables of step 1 were included in logistic regression analyses with early improvement at day 14 as outcome and the significant predictors as criteria."* |
| Riedel et al., 2011 | *"Starting with a logistic regression model including all possible predictors, (all variables with a p-value less than 0.2 in the univariate tests) a forward-backward method based on the lowest Akaike Information Criterion (AIC) was used to identify the relevant predictors to inpatient treatment. The final model was computed with only these predictors and validated using a 10-fold cross-validation with AUC as criterion."* |
| Liang et al., 2023 | *"Following the results of baseline analysis and previous research, (...) were entered into a multinomial logistic regression model. The latent class is the dependent variable. The results can be found in Table 4. Age (χ² = 7.650, P = .022) and baseline score of HAMD (χ² = 161.072, P < .001) were significant predictors. The medications and episode history did not enter the model (supplementary Table 3). The prediction accuracy of the model reached 92.1%."* |
| Rezaei et al., 2021 | *"Moreover, in order to identify the significant predictors of response to tDCS, a binary logistic regression analysis was performed. Independent variables were the cognitive-affective factors of BDI-II as well as different questions of BDI-II (loss of pleasure, agitation, loss of interest, and sleep problems), depression severity (total score of BDI-II at baseline), history of treatment. These variables were chosen due to statistical differences between responders and non-responders at baseline."* |
| Brakemeier et al., 2007 | *"In order to identify significant predictors of response to rTMS, a binary logistic regression analysis was performed. Independent variables were(...). These latter variables were chosen since they were statistically different at baseline between those patients who turned to be responders and those who turned to be non-responders"* |

| Xue et al.,2022 | *"We employed receiver operating characteristic (ROC) curves to estimate the possible predictive value of the hippocampal dFC that indicated significant between-group differences."* |
| --- | --- |
| Nakamura et al., 2021 | *"Based on localization of the significant cluster revealed by the seed‐based exploratory analysis, we focused on the subdivision of the ACC defined by the preceding cortical parcellation study using diffusion tractography (…) the RSFCs‐t at baseline indicated a significant group difference between responders and nonresponders (Mann‐Whitney U‐test, P =.004, Figure 3B). The area under the ROC curve was 0.92 for RSFCs‐t (Figure 3C). "* |
| Xiao et al., 2021 | *"Finally, receiver operating characteristic (ROC) curves were used to characterize the possible predictive value of hippocampal FC that showed significant differences between groups, as well as the summed FC value."* |
| Zhang et al., 2022 & 2023 | zFC values from significant clusters at group level are used for ROC analysis |
| Ye et al., 2022 | *"Lastly, receiver operating characteristic (ROC) curves were carried out to illustrate the possible predictive value of intranetwork FC that showed significant differences between R-MDD and NR-MDD groups."* |
| Hou et al., 2018 | *"The Z-scores of the FC in the bilateral NAcc networks  with significant between-group differences were selected  as potential indicators for the discrimination analysis  (Fig. 4)"* |
| Zhu et al., 2018 | Variable coefficient differences between responders and non responders are used for ROC analysis |
| Hopman et al., 2021 | Identified biomarkers from group-level analysis are combined and used as features in machine learning model |
| Cash et al., 2019 | BOLD signal power from patient/control analysis is used for prediction of treatment outcome. While the labels used for both statistical tests are different, this still consitutes a non-independent selective analysis that may affect results (see Kriegeskorte et al., 2009). |
| Ge et al., 2020 | Identified biomarkers from group-level analysis are used as features in ROC analysis |
| Moreno-Ortega et al., 2019 | *"After multiple comparison correction, decreased connectivity within aDMN(10r), between DLPFC(46) and aDMN(s32), or between DLPFC(p9-46v) and VIS(MT+), remained significant. Even if decreased connectivity within VIS(ventral) did not survive multiple comparison correction, it added predictive value to final models. The main model involved pretreatment RSFC within aDMN(10r) and VIS(ventral), with 100% accuracy of remission within this sample. Leave-one-out cross validation (LOOCV) on FDR corrected models, adjusted by motion displacement regressors, showed 0.83–0.89 prediction accuracy"* |
| Meyer et al., 2019 | Time-invariant main effects of depression recovery are used in machine learning model |
| Crane et al., 2017 | Significant principal components related to treatment outcome in group-level analysis are used in machine learning model |
| Goldstein-Piekarski et al., 2018 | Identified connectivity differences between groups are used in machine learning model while also including clinical features. No model exists on connectivity features alone. |
| Goldstein-Piekarski et al., 2016 | Machine learning model also includes clinical/demographic features. No model exists on imaging features alone |
| Braund et al., 2022 | Functional connectivity features associated with neuroticism in the whole sample are also used to predict treatment outcome |
| Williams et al., 2015 | Amygdala reactivity differences between responders and non-responders at group level are used for sensitivity/specificity analyses |
| Hu et al., 2018 | Differences in hippocampal subvolumes from univariate group-analysis are used for ROC curve analysis |
| Wu et al., 2022 | *"an independent t-test was used to compare the different cortical thickness between the responder group and the non-responder group of SSRI drugs. The average cortical thickness of each significant region was extracted for further group-level analysis, and then ROC curve analysis was performed on the responder group and the non-responder group."* |
| Xu et al., 2022 | *" The predictive models were trained on Dataset 2 and the sensitivity, specificity and accuracy of Dataset 2 were obtained from leave-one-out-cross-validation. Thus, they were not independent and very likely to be inflated."* |
| Cao et al., 2018 | Features used for group-analysis and correlation analyses are included in the machine learning model |
| Korgaonkar et al., 2019 | Machine learning model also includes clinical/demographic features. No model exists on imaging features alone |
