# Supplementary material for: Data leakage in machine learning studies creep into meta-analytic estimates of predictive performance
Source: Mol Psychiatry. 2025 Oct 30;30(12):6070–1. doi: 10.1038/s41380-025-03336-y (PMC12602356; doi:10.1038/s41380-025-03336-y)
Supplement: Supplementary file 2 — Supplementary Figure 1 [file 41380_2025_3336_MOESM2_ESM.docx]

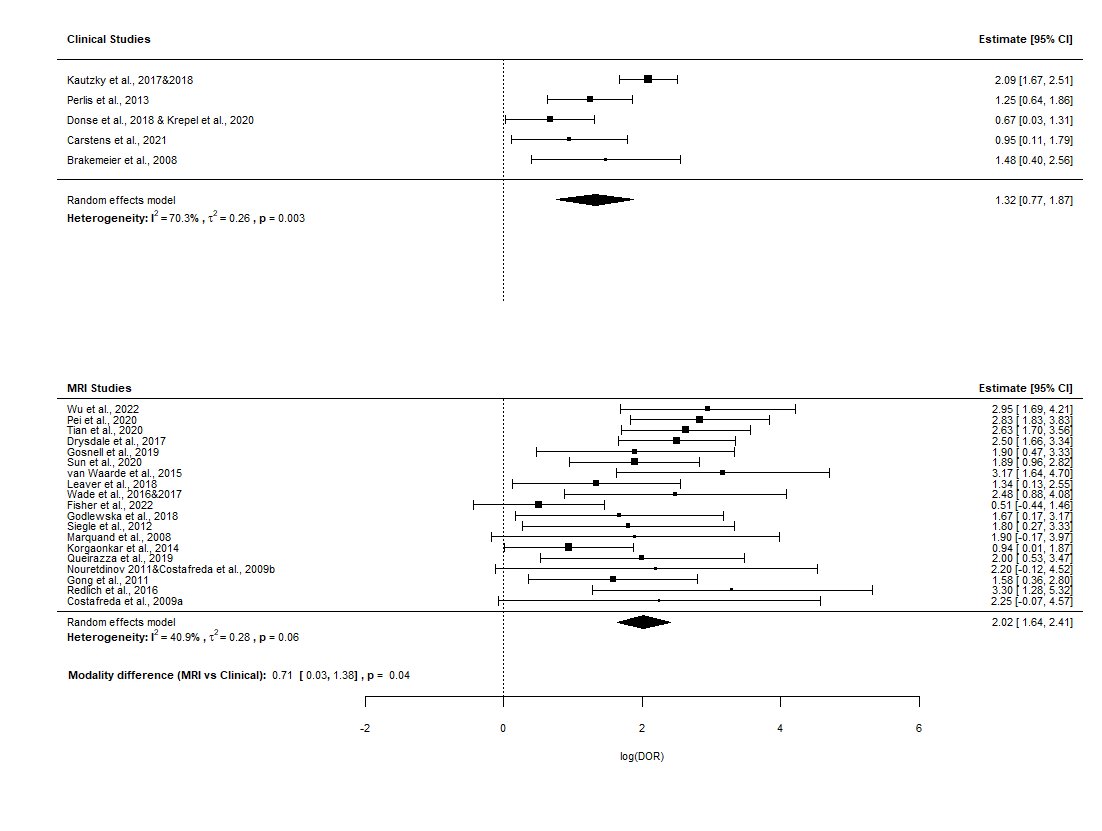
Supplementary Figure 1. Random effects models of the logarithm of diagnostic odds ratio values in the remaining clinical and MRI studies. CI=confidence interval
